# Effect of Training Supervision on Effectiveness of Strength Training for Reducing Neck/Shoulder Pain and Headache in Office Workers: Cluster Randomized Controlled Trial

**DOI:** 10.1155/2014/693013

**Published:** 2014-02-19

**Authors:** Bibi Gram, Christoffer Andersen, Mette K. Zebis, Thomas Bredahl, Mogens T. Pedersen, Ole S. Mortensen, Rigmor H. Jensen, Lars L. Andersen, Gisela Sjøgaard

**Affiliations:** ^1^Institute of Sports Science and Clinical Biomechanics, University of Southern Denmark, 5230 Odense, Denmark; ^2^Institute of Regional Health Research, University of Southern Denmark, 5230 Odense, Denmark; ^3^National Research Centre for the Working Environment, 2100 Copenhagen, Denmark; ^4^Department of Nutrition, Exercise and Sports, University of Copenhagen, 2200 Copenhagen, Denmark; ^5^Department of Occupational Medicine Køge Hospital, 4600 Køge, Denmark; ^6^Danish Headache Center, Department of Neurology, Glosrtup Hospital, University of Copenhagen, 2600 Copenhagen, Denmark

## Abstract

*Objective.* To investigate the effect of workplace neck/shoulder strength training with and without regular supervision on neck/shoulder pain and headache among office workers. *Method.* A 20-week cluster randomized controlled trial among 351 office workers was randomized into three groups: two training groups with the same total amount of planned exercises three times per week (1) with supervision (3WS) throughout the intervention period, (2) with minimal supervision (3MS) only initially, and (3) a reference group (REF). Main outcome is self-reported pain intensity in neck and shoulder (scale 0–9) and headache (scale 0–10). *Results.* Intention-to-treat analyses showed a significant decrease in neck pain intensity the last 7 days in 3MS compared with REF: −0.5 ± 0.2 (*P* < 0.02) and a tendency for 3WS versus REF: −0.4 ± 0.2 (*P* < 0.07). Intensity of headache the last month decreased in both training groups: 3WS versus REF: −1.1 ± 0.2 (*P* < 0.001) and 3MS versus REF: −1.1 ± 0.2 (*P* < 0.001). Additionally, days of headache decreased 1.0 ± 0.5 in 3WS and 1.3 ± 0.5 in 3MS versus REF. There were no differences between the two training groups for any of the variables. *Conclusion.* Neck/shoulder training at the workplace reduced neck pain and headache among office workers independently of the extent of supervision. This finding has important practical implications for future workplace interventions.

## 1. Introduction

Work-related symptoms in neck and shoulder are common among occupational computer users and other sedentary occupations [1, 2] although the evidence of causality is inconclusive [3, 4]. Along with pain in the neck and shoulders, office work is associated with frequent headache and cooccurrence of headache is estimated to be fourfold in workers with musculoskeletal symptoms [5]. Neck pain and headaches are closely related too, although the reported headaches only are rarely diagnosed further into tension-type headache or migraine [6].

Additionally, studies have shown a 31% decrease in quality of life among workers with neck/shoulder symptoms [7] and self-reported health is inversely correlated with neck/shoulder pain [8] and headache [9, 10]. Thus, there is a need for initiatives to reduce the pain problem among office workers who are exposed to repetitive low intensity musculoskeletal load in the neck and shoulder region.

In the past decade, exercise interventions at the workplace have become more common and studies have shown positive effect of physical exercise at work in managing musculoskeletal pain [11–13]. Especially neck pain seems to respond positively to specific strength training, while evidence of strength training impact on shoulder pain is sparse [11–17]. Furthermore, exercise interventions at the workplace have shown significant reduction headache intensity [18] and headache frequency [19].

Exercise programs with supervision are most likely to be beneficial in reducing pain among patients with low back pain [20]. Likewise weekly supervision in maintenance training had significantly better effect than unsupervised training [21], and the effect was closely related to adherence to the program. In a systematic review Coury et al. [12] concluded that there was indication for strong evidence of ineffectiveness for unsupervised training. Exercise programs supervised by instructors enable the participants regularly to tailor the program to the instructions which may have a physiological and motivational value that the unsupervised participants do not benefit from. On the other hand some participants do prefer—after being introduced to the program—to exercise when it fits into their daily routines.

When conducting workplace interventions one may presume that the use of supervision is important to maximize training effects and compliance. Studies that make use of supervision report positive impact on neck/shoulder pain [11, 12, 14–17]; however, the specific effect of the supervision on pain relief is not well established [22]. A study conducting exercise intervention at the workplace showed that only with a single introductory session of supervision a significant reduction of neck/shoulder pain intensity in office workers was attained [23]. However, the study was uncontrolled and did not compare supervised exercises with unsupervised exercises. Supervised training can be expensive and not always an available resource at workplaces. Therefore it is pertinent to reveal the minimum amount of supervision needed for safe and effective exercise training for pain reduction when implementing exercise at the workplaces.

This study is part of a larger intervention program: Workplace adjusted intelligent physical exercise training for reducing musculoskeletal pain in shoulder/neck (VIMS) [24] and investigates the effect of instructor supervised versus minimally supervised exercise training on neck/shoulder pain. The concept of “intelligent physical exercise training” is to balance the individual physiological capacity relatively to occupational exposure, tailor the exercise to individual capacities and disorders, allow for flexibility and personal preferences of the participant, and to be as cost effective for the company as possible.

The aim of this paper is to investigate the relevance of training supervision for safe and effective training, in order to minimize expenses for workplace physical exercise training.

The hypotheses are as follows.

1. Both training interventions have positive effects on neck/shoulder pain and headache compared with reference.
2. Regular supervision of the exercise training will result in a larger effect compared with initial instruction only.
3. Regular supervision of the exercise will have a positive influence on compliance compared with minimal supervision.

## 2. Methods

### 2.1. Study Design

The study was a cluster randomized controlled trial and the intervention period was 20 weeks. The participants were office workers of a national public administrative authority recruited from 12 geographically different units that were located in major cities throughout Denmark.

Randomization was performed on a cluster level to minimize contamination between the participants; for details see Andersen et al. [24]. In short, the clusters were naturally occurring groups of employees working together on a daily basis, being located at the same floor, same office or the like. To ensure the comparability of the training groups and the reference group, the geographical sites were categorized into 13 strata [24]. Adjustments were made in respect to the cluster allocation due to 26 participants being relocated to other work sites between the time of randomization and the start-up of the different interventions (approx. 3 weeks) in order to have these participants follow the intervention for the cluster of their new colleagues. No subsequent reallocations were performed. The participants were randomized into five groups: one reference group (REF) without exercise training and four training groups performing specific strength training. The present study addressed only two of these training groups: one was scheduled for training 3 × 20 minutes per week with supervising half of the sessions throughout the training period (3WS) and the other group was likewise scheduled for training 3 times per week but only received minimal supervision (3MS), which was given initially in terms of instructions for 2 sessions to learn the exercises correctly. The total number of planned training was 60 sessions (3 × 20 weeks) of which 3WS had instructors supervising the training 10 hours (30 sessions × 20 min), while 3MS had instructor supervision for 40–60 min (due to absence by some participants instructors would usually have to come for 2-3 training sessions for this group). A previous paper addresses the other training groups [14].

Written informed consent was obtained from all participants before they entered the study.

The study protocol was approved by the local ethics committee (H-C-2008-103) and registered in ClinicalTrials.gov (no. NCT01027390).

### 2.2. Participants

About half of the participants were recruited from the Capital Region and the other half from other parts of Denmark. Thereby the population is nationally representative and strengths external validity. The eligibility criterion was employees performing office work for at least half of their working hours. The exclusion criteria were (i) hypertension (systolic BP > 160, diastolic BP > 100) or cardiovascular diseases, (ii) symptomatic herniated disc or severe disorders of the cervical spine, (iii) postoperative conditions in the neck or shoulder region, (iv) history of severe trauma, (v) pregnancy, (vi) or serious disease.

### 2.3. Intervention

The two training groups had the same total amount of exercises and repetitions planned three times per week. The training groups performed specific strength training with 4 different dumbbell exercises for the neck and shoulder muscles and one for the wrist as described in detail previously [24]: front raise, lateral raise, reverse flies, and shrugs.

The participants performed warm-up exercises in the beginning of each training session (10 repetition of each exercise with 50% of 1 repetition maximum (RM)). At the beginning and halfway through the intervention period, the participants were tested for optimizing the training intensity and the loads were progressively increased according to the principle of periodization and progressive overload [25]. The intensity of the program increased gradually from 20 RM at the beginning of the intervention period to 8 RM further along in the process.

### 2.4. Outcome Measures

Structured e-mail based questionnaires were applied before and after the intervention. The primary outcome was musculoskeletal pain symptoms in neck/shoulder and secondary outcome was headache characteristics [24].

The standardized nordic questionnaire [26] was applied at baseline before the randomization and repeated after the intervention. The questions were “How many days have you had trouble in body part during the last three months?” (0 days; 1–7 days; 8–30 days; >30 days; everyday) for symptom duration, and “On average, how intense was your pain in body part during the last three months on a scale ranging from 0 to 9?” where 0 is no pain and 9 is worst imaginable pain for symptom intensity. The same question was also asked for pain during the previous seven days.

Secondary outcome variables were headache characteristics (frequency and pain intensity).

Question about duration of headache was “How many days have you had a headache during the previous month?” The following response options were 0, 1–3, 4–7, 8–14, and >14 days. For subsequent analyses 1–3 days were recorded to 2 days, 4–7 days to 5.5 days, 8–14 to 11 days, and >14 to 20 days. Intensity of headache was also inquired about “On average, how bad were your headaches when you experienced them during the previous month?” where 0 is no pain and 10 is worst imaginable pain [19].

Compliance was based on follow-up questionnaire replies on training frequency (completers).

The response categories were  (1)  “regular exercise training 40–60 min/week,”  (2)  “regular exercise training 20–40 min/week,”  (3)  “not regular but at least 80 min/month,” and  (4)  “not regular but at least 40–60 min/month.” Regular training was collapsed into “regular exercise training 20–60 min per week.”

### 2.5. Statistical Analyses

The statistical analyses were based on an intention-to-treat approach (ITT) via Stata SE12 (StataCorp LP, College Station, Texas). Missing values in postmeasurements were substituted with the last observation carried forward [27, 28]. Differences between groups in neck/shoulder pain and headache frequency and intensity were tested using analyses of covariance (ANCOVA) with the level at baseline and sex as a covariate.

In addition to the ITT analyses we performed analyses using ANCOVA only on completers, defined as those who had answered the questionnaire before and after the intervention and the rest were defined as noncompleters. Relationship between neck pain and headache was estimated using Spearman rank correlation. We also defined a subgroup, neck-pain cases, as those who at baseline reported pain intensity in the neck during the last 3 months of 3 or more (scale 0–9) [29]. This sub group analysis was performed on ITT data as well as completers only.

Results were considered statistically significant if the 2-tailed *P* value was ≤ 0.05.

## 3. Results

### 3.1. Baseline

**Figure 1:**
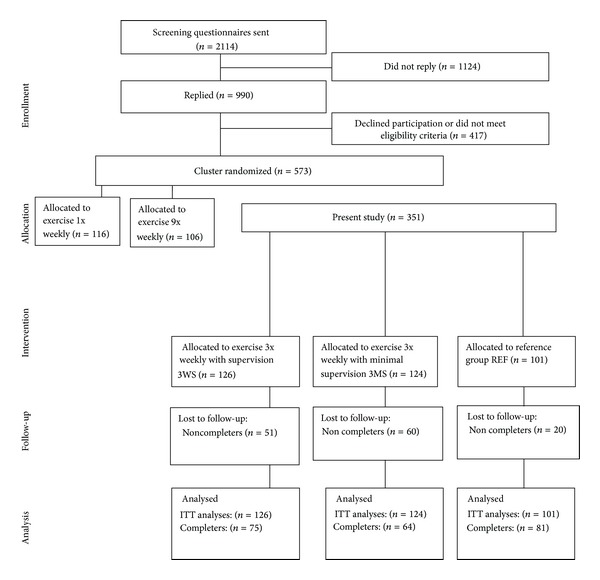
Flowchart of the trial.

Flow of participants through the trial is presented in Figure 1. The present study included 351 participants cluster randomized in 3 groups: 3WS (*n* = 126), 3MS (*n* = 124), and REF (*n* = 101). At baseline there were no significant differences between the groups (Table 1).

Baseline data on demographics and pain variables for the entire study group is presented in Table 1 and for completers and neck-pain cases in Table 2. Analyses on completers (*n* = 220) versus noncompleters (*n* = 131) showed no significant differences between these groups at baseline on pain variables. However, noncompleters were significantly younger than completers; mean age 44 ± 1.4 versus 47 ± 0.7 (*P* < 0.05).

Four participants reported nonpermanent injuries during the intervention period: back pain (*n* = 2), shoulder/wrist pain (*n* = 1), and pain in the knee (*n* = 1).

Mean values on neck pain were ~3 on a scale 0–9 (Table 1) and neck-pain cases accounted for 56% (pain intensity 3 or more the last 3 months). Of note is further that relatively many participants reported pain intensity corresponding to 4 or more: 41% (the last 3 months) and 32% (the last 7 days).

Regarding headache, approximately 15% of the participants reported having headache above 7 days the previous month with an average intensity at 7.0 ± 1.9. Average number of days in which participants used medication because of headache was for WS: 2.3 ± 1.0, MS: 2.2 ± 1.0, and REF: 2.4 ± 0.9.

Among neck-pain cases (*n* = 197), 90% also reported headache; mean headache frequency at baseline is 6.5 ± 5.9 days of previous month.

A statistical significant relationship between intensity of neck pain the last three months and intensity of headache during the previous month was identified (Spearman correlation, *r*:  0.39 (*P* < 0.001)).

### 3.2. Intervention

#### 3.2.1. Primary Outcome

*Neck and Shoulder Pain (ITT analyses).* Intention-to-treat analyses showed a significant decrease in neck pain intensity the last 7 days in 3MS compared with REF: −0.5 ± 0.2 (*P* < 0.02) and a tendency for 3WS versus REF: −0.4 ± 0.2 (*P* < 0.07) (Table 3). Analyses on neck pain the last 3 months and shoulder pain did not show any significant changes (Table 3).

ITT-analyses for the group defined as neck-pain cases (*n* = 197) showed a significant decrease in neck pain the last 7 days, 3WS versus REF: −0.7 ± 0.4 (*P* < 0.05) but not for 3MS versus REF. Similarly, analyses on neck pain over the last 3 months showed significant decrease in neck pain only in 3WS versus REF: −0.7 ± 0.4 (*P* = 0.05). There were no significant changes in shoulder pain within the group of neck-pain cases.

*Neck and Shoulder Pain (completers). *In both training groups there were significant decreases in the intensity of neck pain (last 3 months): 3WS versus REF: −1.0 ± 0.3 (*P* < 0.001) and 3MS versus REF: −0.9 ± 0.3 (*P* < 0.001) (Table 4). Additionally, both groups showed a significant decrease in the intensity of neck pain (7 days): 3WS versus REF: −1.0 ± 0.3 (*P* < 0.001) and 3MS versus REF: −1.1 ± 0.3 (*P* < 0.001). The same applied to the intensity of shoulder pain the last 3 months in both training groups: 3WS versus REF: −0.7 ± 0.3 (*P* < 0.01) and 3MS versus REF: −0.6 ± 0.3 (*P* < 0.05). There were no significant changes in the intensity of shoulder pain the last 7 days (Table 4).

At baseline, completers defined as neck-pain cases (*n* = 124) had intensity of neck pain (last 3 months) corresponding to 5.1 ± 1.6 (3WS), 5.2 ± 2.0 (3MS), and 4.1 ± 2.1 (REF). Further censored analyses on this sub-group showed significant changes in the intensity of neck pain the last 3 months for both training groups compared to REF: 3WS: −1.9 ± 0.4 (*P* < 0.001) and 3MS: −1.1 ± 0.5 (*P* < 0.03) as well as in intensity of neck pain the past 7 days: 3WS: −1.7 ± 0.5  (*P* < 0.001) and 3MS: −1.4 ± 0.5 (*P* < 0.004) (Table 5). Furthermore, among completers defined as neck-pain cases there was a significant difference between 3WS and 3MS in intensity of neck pain (last 3 months) with better improvement in 3WS; 0.8 ± 0.4 (*P* = 0.05). Concerning shoulder pain within this sub-group there was a significant decrease in intensity of shoulder pain the last 3 months in the 3WS group compared to the reference group: −1.2 ± 0.4 (*P* < 0.003) (right shoulder) and −0.8 ± 0.4 (*P* < 0.03) (left shoulder) but not in the 3MS group (Table 5). There were no significant changes in intensity of shoulder pain the last 7 days, neither in 3WS nor in 3MS.

#### 3.2.2. Secondary Outcome

*Headache (ITT analyses)*. Results of secondary outcome variables are shown in Table 3. There was a significant reduction in days with headache in both training groups compared to the reference group. Furthermore, there was a significant decrease in pain intensity in both training groups compared to the reference group. In the group of neck-pain cases there was a significant decrease in headache intensity in 3WS versus REF: −0.9 ± 0.4 (*P* < 0.02) and 3MS versus REF: −0.9 ± 0.3 (*P* < 0.01).

After the intervention, there were no changes in the use of medication because of headache.

*Headache (completers). *Days with headache last month decreased significantly in both groups compared to the reference group: 3WS versus REF: −0.6 ± 0 (*P* < 0.001) and 3MS versus REF: −0.6 ± 0.1 (*P* < 0.001), and the pain intensity decreased significantly in both training groups compared to the reference group: 3WS versus REF: −1.6 ± 0.4 (*P* < 0.00) 1 and 3MS versus REF: −1.5 ± 0.3 (*P* < 0.001) (Table 4).

*Compliance. *Among completers 60% in the 3WS group and 47% in the 3MS group reported that they were exercising on a regular basis 20–60 min a week in the intervention period. There was no significant difference in compliance between the groups (*P* < 0.14) with an overall value of 54% participating at a regular basis.

Regarding primary and secondary outcomes, the analyses on the entire group showed no significant difference between the two training groups.

## 4. Discussion 

The major findings of this study were significant reductions of similar magnitude in neck/shoulder pain and in headache for 3WS and 3MS compared to REF after a 20-week training period. Furthermore, the training program was considered to be safe since only 4 out of 351 participants (1%) reported transitory adverse events of short duration.

Our study hypothesis was that training with supervision would be more effective on neck/shoulder pain as well as headache reduction and that the supervision would cause better compliance than training without supervision. The study could not confirm this hypothesis since the sizes of these effects of pain reduction among the intervention groups were of the same order of magnitude. By and large, the relative difference between baseline and postmeasurements was 10–20% for neck pain and ~30% for headache intensity for both training groups in the ITT analyses when compared to REF. Similarly, the relative difference was ~30–40% for neck pain and ~30% for headache intensity among completers. In the group of pain cases the relative difference was 16–8% for headache intensity.

The results showed relatively high intensity of neck pain since approx. 40% of the participants reported neck pain corresponding to 3 or above 3 on a scale 1–10. Furthermore, relatively high frequency of days with headache was reported at baseline.

Reduction of neck pain associated with work site intervention program in this study is consistent with results from previous studies [11–15] and confirms also the positive effect of exercise training to reduce headache [17, 18, 30]. In this study, the headache could not be further classified or quantified into tension-type headache or migraine, as it required a detailed diagnostic interview and a neurological examination plus a prospective diagnostic diary which is quite time consuming and complicated to be applied in large scale working place studies like the present.

It is highly relevant to investigate the importance of training supervision when conducting exercise training at the workplace because recruitment of training instructors is elaborative and expensive and may result in restriction of training times. Training supervision as used in present study requires an annual basis salary of approx. 78 hours for instructors. This may hinder implementation of exercise training programs during working hours. Based on the scientific literature the effect of supervision shows conflicting findings. Zavanela et al. [31] showed decreased neck/shoulder pain and headache of bus drivers after 24 weeks of supervised training intervention. On the other hand, Mongini et al. [32] reported decrease in neck/shoulder pain and headache in a large randomized controlled trial using an unsupervised program. However, none of these two studies were designed to measure the effect of supervision compared to no supervision. Interestingly, ITT analyses in the present study for the group defined as neck-pain cases showed significant decrease in neck pain the last 7 days and the last 3 months in the group with training supervision (3WS) but not for the minimally supervised group (3MS). Thus, we cannot exclude that the pain condition for participants may influence the need for supervision such that patients or those in pain to a larger extend benefit from proper instruction than pain-free participants. To evaluate the effect of the supervision, compliance is crucial. Forty percent of the 3WS group, 48% of 3MS group, and 20% of the REF did not answer the questionnaire after the intervention. This is limiting factor for the study and might induce type 2 error.

Only 60% of completers in the 3WS group and 47% in the 3MS group reported that they were exercising on a regular basis in the intervention period, which may be a limitation when evaluating the effect of the supervision. The reason for these levels of participation may be that supervision is motivating but also causing time constrains, while training without supervision gives more flexibility but may lack motivating actions.

Since noncompleters did not return the questionnaire, we are not able to conclude upon reasons behind not responding on the second questionnaire or upon questions regarding supervision. Noncompleters were in this study defined as those who did not reply the second questionnaire, but as a term, noncompleter is not unambiguous. Although the final questionnaire is not completed, participants could, in principle, have been training a large part of the intervention period. Ongoing evaluation of the included population and a detailed interview could possibly elucidate their training process and outcome.

Dropout and poor compliance are always a challenge in intervention studies [11, 15, 33] and balanced strategies to maintain long-term motivation in studies with exercises interventions are pertinent [17, 18].

## 5. Conclusion

One hour of physical exercise training per week for 20 weeks at the workplace was highly effective to reduce neck pain and headache and the effect was overall independent of the level of supervision. A well-performed introduction and supervision of the exercises only in the beginning of the relatively simple training program was sufficient to achieve pain-relieving effects. Greater flexibility in planning and conducting exercise training at the workplace due to no constraint with supervised training schedules may have advantages both for the employees and for the employer at a lower cost compared to supervised training.

## Figures and Tables

**Table 1:** Baseline demographic and clinical characteristics of trial groups.

Characteristics	Exercise group 3WS (*n* = 126)	Exercise group 3MS (*n* = 124)	Reference group (*n* = 101)	Min–Max (*n* = 351)	*P*
Sex, (m/f)	39/87	52/72	42/59		NS
Age, (y)	46 ± 10	45 ± 11	46 ± 10	22–66	NS
BMI	24.7 ± 4.3	25.6 ± 3.8	26.0 ± 4.5	14–45	NS
Pain (on scale 0–9)					
Neck pain (3 months)	3.1 ± 2.4	3.2 ± 2.4	3.2 ± 2.3	0–9	NS
Neck pain (7 days)	2.6 ± 2.5	2.4 ± 2.4	2.5 ± 2.5	0–9	NS
Right shoulder pain (3 months)	2.3 ± 2.4	2.0 ± 2.4	2.0 ± 2.4	0–8	NS
Right shoulder pain (7 days)	1.8 ± 2.3	1.6 ± 2.2	1.6 ± 2.3	0–8	NS
Left shoulder pain (3 months)	1.8 ± 2.3	1.6 ± 2.3	1.5 ± 1.9	0–9	NS
Left shoulder pain (7 days)	1.4 ± 2.0	1.3 ± 2.0	1.3 ± 1.9	0–8	NS
Headache (pain scale 0–10)	3.4 ± 2.5	3.6 ± 2.8	3.6 ± 3.0	1–10 (*n* = 211)	NS
Headache (days of last month)	3.8 ± 4.3	4.1 ± 4.7	4.2 ± 4.9	0–>14 days	NS

Values are mean (SD) and numbers. *P* values for the 1-way analysis of variances.

**Table 2:** Baseline neck pain, right shoulder pain, and headache in completers and neck-pain cases, respectively.

Characteristics	Exercise group 3WS		Exercise group 3MS		Reference group		*P*
	Completers (*n* = 75)	Neck-pain cases (*n* = 69)	Completers (*n* = 64)	Neck-pain cases (*n* = 70)	Completers (*n* = 81)	Neck-pain cases (*n* = 58)	
Completers (neck-pain cases)							
Neck pain (3 months)	3.0 ± 2.5	5.0 ± 1.6	3.5 ± 2.5	4.9 ± 1.7	3.4 ± 2.3	4.8 ± 1.6	NS
Neck pain (7 days)	2.4 ± 2.5	4.2 ± 2.1	2.5 ± 2.4	3.8 ± 2.3	2.7 ± 2.5	3.9 ± 2.2	NS
Right shoulder pain (3 months)	1.8 ± 2.2	3.3 ± 2.4	2.1 ± 2.4	2.7 ± 2.6	1.9 ± 2.4	3.2 ± 2.5	NS
Right shoulder pain (7 days)	1.3 ± 1.9	2.8 ± 2.5	1.5 ± 2.2	2.2 ± 2.4	1.6 ± 2.3	2.7 ± 2.6	NS
Left shoulder pain (3 months)	1.4 ± 2.0	2.8 ± 2.5	1.8 ± 2.4	2.2 ± 2.3	1.6 ± 2.0	2.2 ± 2.2	NS
Left shoulder pain (7 days)	1.0 ± 1.7	2.2 ± 2.3	1.4 ± 2.1	2.0 ± 2.3	1.5 ± 2.1	2.0 ± 2.2	NS
Headache (pain scale 0–10)	4.9 ± 1.8	5.5 ± 2.0	5.5 ± 2.8	6.1 ± 2.4	5.7 ± 2.5	6.4 ± 2.5	NS
Headache (days last month)	2.2 ± 1.0	2.6 ± 1.1	2.5 ± 2.8	2.8 ± 1.2	4.4 ± 1.1	2.8 ± 1.2	NS

Values are mean (SD) and numbers. *P* values for the 1-way analysis of variances. Completers had answered the questionnaire before and after the intervention. Neck-pain cases reported pain intensity in neck last 3 months of 3 or more at baseline (scale ranging from 0 to 9).

**Table 3:** Summary results for each study group after 20 weeks of intervention (ITT data).

Characteristics	3WS Post-pre (SD) (*n* = 126)	3MS Post-pre (SD) (*n* = 124)	Ref. group Post-pre (SD) (*n* = 101)	Difference 3WS versus REF (95% CI) (SE)	*P*	Difference 3MS versus REF (95% CI) (SE)	*P*
Pain (a scale ranging from 0 to 9)							
Neck pain (3 months)	−0.9 ± 2.1	−0.9 ± 1.5	−0.6 ± 2.0	−0.4 ± 0.2 (−0.8 to 0.1)	0.11	−0.3 ± 0.2 (−0.7 to 0.1)	0.15
Neck pain (7 days)	−0.7 ± 2.1	−0.6 ± 1.5	−0.2 ± 2.0	−0.4 ± 0.2 (−0.9 to 0.03)	0.07	−0.5 ± 0.2 (−0.9 to −0.1)	0.02*
Right shoulder pain (3 months)	−0.5 ± 1.9	−0.5 ± 2.0	−0.2 ± 1.9	−0.1 ± 0.2 (−0.6 to 0.3)	0.50	−0.3 ± 0.2 (−0.8 to 0.1)	0.13
Right shoulder pain (7 days)	−0.3 ± 1.8	−0.4 ± 1.8	−0.2 ± 1.9	0.0 ± 0.2 (−0.4 to 0.4)	0.97	−0.2 ± 0.2 (−0.6 to 0.3)	0.43
Left shoulder pain (3 months)	−0.4 ± 1.5	−0.5 ± 1.5	−0.3 ± 1.8	0.0 ± 0.2 (−0.4 to 0.4)	0.99	−0.2 ± 0.2 (−0.5 to 0.2)	0.46
Left shoulder pain (7 days)	−0.8 ± 1.6	−0.3 ± 1.4	−0.4 ± 1.8	0.3 ± 0.2 (−0.1 to 0.7)	0.19	0.1 ± 0.2 (−0.3 to 0.4)	0.77
Headache (pain scale, 0–10)	−0.4 ± 1.8	−0.4 ± 1.4	0.7 ± 2.2	−1.1 ± 0.2 (−1.6 to −0.6)	0.00*	−1.1 ± 0.2 (−1.5 to −0.6)	0.00*
Headache (days of last month)	−0.4 ± 3.7	−0.7 ± 2.6	0.6 ± 4.4	−1.1 ± 0.5 (−2.1 to −0.1)	0.03*	−1.3 ± 0.5 (−2.2 to −0.5)	0.00*

Changes in post-pre values are absolute and not adjusted. Differences are estimated as the difference between means, with 95% confidence intervals, based on the 1-factor analyses of covariance with the level at baseline and sex as a covariate. *Significant change.

**Table 4:** Summary results for each study group after 20 weeks of intervention (completers).

Characteristics	3WS: post-pre (SD) (*n*)	3MS: post-pre (SD) (*n*)	Ref.: post-pre (SD) (*n*)	Difference 3WS versus REF (95% CI) (SE)	*P*	Difference 3MS versus REF (95% CI) (SE)	*P*
Neck pain (3 months)	−1.5 ± 2.5 (75)	−1.8 ± 1.7 (64)	−0.7 ± 2.2 (81)	−1.0 ± 0.3 (−1.5 to −0.4)	0.00*	−0.9 ± 0.3 (−1.5 to −0.4)	0.00*
Neck pain (7 days)	−1.1 ± 2.6 (75)	−1.2 ± 1.9 (64)	−0.2 ± 2.2 (81)	−1.0 ± 0.3 (−1.6 to −0.4)	0.00*	−1.1 ± 0.3 (−1.6 to −0.5)	0.00*
Right shoulder pain (3 months)	−0.8 ± 2.4 (75)	−1.0 ± 2.7 (64)	−0.2 ± 2.1 (81)	−0.7 ± 0.3 (−1.2 to −0.2)	0.01*	−0.6 ± 0.3 (−1.2 to 0.0)	0.04*
Right shoulder pain (7 days)	−0.5 ± 2.3 (75)	−0.7 ± 2.5 (64)	−0.2 ± 2.1 (81)	−0.5 ± 0.3 (−1.0 to 0.0)	0.07	−0.5 ± 0.3 (−1.1 to 0.1)	0.09
Left shoulder pain (3 months)	−0.7 ± 1.9 (75)	−0.9 ± 2.0 (64)	−0.4 ± 2.0 (81)	−0.0 ± 0.2 (−0.5 to 0.4)	0.89	0.4 ± 0.2 (−0.1 to 0.9)	0.10
Left shoulder pain (7 days)	−0.3 ± 2.1 (75)	−0.6 ± 1.9 (64)	−0.5 ± 2.0 (81)	−0.2 ± 0.3 (−0.7 to 0.4)	0.59	0.0 ± 0.3 (−0.5 to 0.6)	0.90
Headache (pain scale 0–10)	−2.0 ± 2.2 (58)	−2.1 ± 1.6 (55)	−0.7 ± 2.2 (64)	−1.6 ± 0.4 (−2.4 to −0.8)	0.00*	−1.5 ± 0.3 (−2.2 to −0.9)	0.00*
Headache (days of last month)	−0.6 ± 4.9 (75)	−1.4 ± 3.6 (64)	−0.7 ± 4.9 (81)	−1.9 ± 0.7 (−3.3 to −0.5)	0.01*	−2.2 ± 0.6 (−3.4 to −1.0)	0.00*

Changes in post-pre values are absolute and not adjusted. Values are means with standard deviation presented for each group (completers). Differences are estimated as the difference between means (SE), with 95% confidence intervals, based on the 1-factor analyses of covariance with the level at baseline and sex as a covariate. *Significant change.

**Table 5:** Summary results for each neck-pain cases group after 20 weeks of intervention (completers only).

Characteristics	3WS: post-pre (SD) (*n* = 39)	3MS: post-pre (SD) (*n* = 37)	Ref.: post-pre (SD) (*n* = 48)	Difference 3WS versus REF (95% CI) (SE)	*P*	Difference 3MS versus REF (95% CI) (SE)	*P*
Neck pain (3 months)	−3.1 ± 2.2	−2.4 ± 1.9	−1.2 ± 2.6	−1.9 ± 0.4	0.000*	−1.1 ± 0.5	0.022*
Neck pain (7 days)	−2.4 ± 2.7	−1.8 ± 2.1	−0.6 ± 2.5	−1.7 ± 0.5	0.000*	−1.4 ± 0.5	0.003*
Right shoulder pain (3 months)	−1.7 ± 2.5	−1.3 ± 3.2	−0.7 ± 2.4	−1.1 ± 0.4	0.002*	−0.8 ± 0.5	0.096
Right shoulder pain (7 days)	−1.2 ± 2.8	−0.9 ± 2.9	−0.7 ± 2.5	−0.8 ± 0.4	0.062	−0.7 ± 0.5	0.170
Left shoulder pain (3 months)	−1.4 ± 2.2	−1.4 ± 2.2	−0.6 ± 2.2	−0.8 ± 0.4	0.024*	−0.6 ± 0.4	0.156
Left shoulder pain (7 days)	−0.8 ± 2.6	−1.0 ± 2.3	−0.9 ± 2.4	−0.2 ± 0.4	0.634	−0.2 ± 0.4	0.727
Headache (pain scale 0–10)	−0.5 ± 2.8	−0.9 ± 1.5	0.5 ± 2.5	−1.4 ± 0.5	0.008*	−1.3 ± 0.4	0.001*
Headache (days of last month)	−0.9 ± 4.7	−1.4 ± 3.1	0.9 ± 4.6	−2.3 ± 0.9	0.010*	−2.1 ± 0.8	0.006*

Changes in post-pre values are absolute and not adjusted. Values are means with standard deviation presented for each group (neck-pain and completers). Differences are estimated as the difference between means (SE), with 95% confidence intervals, based on the 1-factor analyses of covariance with the level at baseline and sex as a covariate. *Significant change.
